# Managing patients’ reports of abdominal pain and irritable bowel syndrome-like symptoms during quiescent inflammatory bowel disease: a role for shared sensemaking

**DOI:** 10.1177/20494637241230807

**Published:** 2024-01-29

**Authors:** Danielle Huisman, Felice Fernhout, Faye Moxham, Christine Norton, Kirsty Bannister, Rona Moss-Morris

**Affiliations:** 1 Health Psychology Section, Institute of Psychiatry, Psychology and Neuroscience, 34426 King’s College London, London, UK; 2 Florence Nightingale Faculty of Nursing, Midwifery and Palliative Care, 14272 King’s College London, London, UK; 3 Central Modulation of Pain, Institute of Psychiatry, Psychology and Neuroscience, 34426 King’s College London, London, UK

**Keywords:** inflammatory bowel disease, irritable bowel disease, abdominal pain, remission, qualitative research

## Abstract

**Background:**

Patients with inflammatory bowel disease (IBD) are often faced with distressing and confusing abdominal pain during remission. Some people respond adversely to healthcare professionals’ (HCPs) suggestions that this pain and related symptoms are due to secondary irritable bowel syndrome (IBS). Exploring how HCPs view, manage, and explain pain during quiescent disease may provide insights into how communication can be improved to increase understanding and mitigate negative responses.

**Methods:**

In-depth semi-structured interviews were conducted with 12 IBD-nurses (*n* = 4) and gastroenterologists (*n* = 8) working in the United Kingdom or the Netherlands. Reflexive thematic analysis was used to analyse interviews.

**Results:**

Findings suggest that HCPs pay relatively little attention to pain when there is no underlying pathology and prefer to concentrate on objectifiable causes of symptoms and treating disease activity (*Theme 1: Focus on disease activity, not pain and associated symptoms*). Explanations of abdominal pain and IBS-like symptoms during remission were not standardised (*Theme 2: Idiosyncratic and uncertain explanations for pain during remission*). Processes of shared decision-making were outlined and shared sensemaking was reported as a strategy to enhance acceptance of IBS explanations (*Theme 3: Shared decision making versus shared sensemaking*).

**Conclusion:**

Future work should focus on establishing how pain during remission may be best defined, when to diagnose IBS in the context of IBD, and how to explain both to patients. The formulation of standardised explanations is recommended as they might help HCPs to adopt practices of shared sensemaking and shared decision-making. Explanations should be adaptable to specific symptom presentations and different health literacy levels.

## Introduction

Inflammatory bowel disease (IBD) describes relapsing and remitting conditions, including Crohn’s disease and ulcerative colitis, where maladaptive immune responses culminate in inflammation of the digestive system.^1^ Abdominal pain is common in these conditions and does not necessarily abate during periods of remission.^2^ Findings from a recent interview study with IBD patients suggests that ongoing abdominal pain, alongside other symptoms, is often distressing and creates uncertainty about the actuality of remission, especially in the period following diagnosis.^3^

Disease activity is investigated by history taking, physical examination, symptom assessment – aided by clinical tools such as the Harvey Bradshaw Index (HBI) and the Simple Clinical Colitis Activity Index (SCCAI)^4,5^ – and biochemical assessment – using measurements of C-reactive protein and/or faecal calprotectin.^6^ If needed, a series of more complex endoscopic, histologic, and/or radiographic investigations can be done. When disease activity is ruled out, patients are sometimes told by healthcare professionals (HCPs) that they have secondary irritable bowel syndrome (IBS). IBS refers to a brain-gut disorder affirmed by certain symptoms alongside the elimination of medical causes (a positive symptom approach).^7^

In the NICE definition, the primary symptom of IBS is the experience of abdominal pain or discomfort in combination with alterations in stool habit for at least six months and the absence of alarm symptoms or signs.^8^ The stricter Rome IV criteria, often regarded as the gold standard, do not include abdominal discomfort and require people to have abdominal pain at least one day a week over a period of three months.^7,9,10^ Approximately 23% of people with IBD meet the Rome criteria for IBS when remission is established endoscopically.^11^ Diagnosing secondary IBS may help tailor therapy and prevent unnecessary treatment with immunosuppressants.^12^ However, the role of IBS in IBD remains contested as IBS criteria are not validated for people with IBD. ^11,13^

In a recent qualitative study, patients we interviewed were often unclear as to why their abdominal pain endured during remission. An IBS diagnosis did not necessarily help them comprehend (and manage) ongoing pain better as they were seldomly accompanied with explanations (or treatment), and some found an IBS label for their symptoms unacceptable.^3^ In this study, we wanted to better understand HCPs’ perceptions of abdominal pain and IBS-symptoms during quiescent IBD. We know that patients and HCPs do not always understand IBD flare and remission in the same way.^14^ A richer understanding of HCPs’ viewpoints in this area may help find ways to mitigate negative responses and facilitate communication that leads to clearer understanding of abdominal pain during remission and ways to manage these.^3^ Our objectives were three-fold: to explore HCPs perceptions of abdominal pain and secondary IBS in IBD during quiescent disease, their considerations when communicating these to patients with IBD, and what factors underlie (the mode of) communication

## Methods

We conducted in-depth interviews with a loose structure and open-ended questions. Ethical approval was obtained from the University Research Ethics Committee of King’s College London (MRSP-20/21-22685).

### Participants

We used networks of people in the field to reach out to IBD-nurses and gastroenterologists working in the United Kingdom or the Netherlands. Participants currently offered care to patients with IBD and spoke either English or Dutch.

### Procedure

Interviews were conducted in English or Dutch by DH and recorded with a secure videoconference app (Microsoft Teams, Version 1.4.00.4167). A topic guide of core questions was developed (see Table 1) and agreed by the wider research team. Interviews and analysis were performed iteratively allowing analysis to inform data collection.^15^ When the researchers felt they had approached thematic saturation, recruitment was ceased.^16,17^

**Table 1.** Topic guide.

Questions	Probes
1. How is pain managed in your service? Do you have a particular role in this?	a) What role do you think IBD clinicians play in the context of pain in IBD? For example, management? Communication? b) Do you feel that the pandemic has impacted management of pain in IBD in any way? How?
2. Can you tell me a bit about what you tell patients about abdominal pain in IBD?	a) How do you view pain in active disease versus remission? b) What do you say to patients who continue to experience pain during remission? What are the difficulties/challenges when you discuss pain during remission? c) What, in your perception, do patients find helpful when discussing pain during remission? What do they find less helpful? d) How do you adapt what you say to specific patients? What are reasons to adapt? e) Has what you tell patients changed during COVID? How?
3. How do you see IBS in relation to IBD?	a) Do you bring up IBS? When? What do you say? b) How do people respond to you bringing up IBS? How do you manage an aversive response? c) How do you respond to patients bringing up IBS?
4. What resources (e.g. websites) would you recommend to patients to find out more about IBD?	a) Are there specific ones that you think are particularly good for people with pain? People with ongoing symptoms? b) Are there any IBS resources that you would recommend?
5. I would like to ask some very short questions, before we finish	a) What is your age? b) How many years have you been working with IBD patients? c) Do you solely see IBD patients? d) What other patients do you see? e) Do you see IBS patients? f) If you do not mind me asking, what is your ethnicity?
6. Is there anything else you would like to add before we finish?	a) n/a

Participant information sheets were posted online and emailed to HCPs. Those who agreed to participate were sent a link to an informed consent form generated using Qualtrics software (Qualtrics, Provo, UT). Audio-records of the English interviews were anonymised and transcribed verbatim by a professional transcriber, Dutch interviews were transcribed by FF.

### Analysis

This study used reflective thematic analysis to analyse the interviews.^18–20^ This form of analysis regards the subjectivity and reflexivity of the researchers as assets.^20^ DH, FF, and FM divided the transcripts between themselves: the first nine transcripts were independently coded by two researchers, the last three were done by DH alone. Analysis progressed in six stages.^18^ Firstly, the researchers familiarised themselves with the data by closely reading the transcripts and listening to the interviews (DH did so for all the interviews, while FF and FM focussed on those allotted to them). Secondly, the allotted transcripts were coded line-by-line, on paper, to establish an early level of abstraction. Thirdly, transcripts were entered into NVIVO (version 12), and independently developed codes were compared, discussed, refined, inputted, and collated into potential themes. Fourthly, the themes were compared against the quotes and dataset to ensure that they reflected the source material. Fifthly, themes and definitions were refined allowing for a concise rapport of findings (stage 6).

## Results

Demographic characteristics of participants (*n* = 12) are shown in Table 2. Eight were gastroenterologists and four IBD-nurses (five and three practicing in the UK, respectively). Half the sample saw IBD patients exclusively. Interviews lasted 62 minutes on average (range 44–96 minutes) and were conducted between May 2021 and June 2022. We generated three themes; theme 1, *Focus on disease activity, not pain and associated symptoms,* outlines the parameters by which to understand the intricacies of pain explanations described in theme 2, *Idiosyncratic and uncertain explanations for pain during remission*, and theme 3, *Shared decision-making versus shared sensemaking*.

**Table 2.** Sample characteristics (*n* = 12) expressed in numbers and percentages unless stated otherwise.

Socio-demographics	
Females	5 (41.6%)
Age in years, mean (SD)	41 (9.4)
Ethnicity	
White	6 (50.0%)
Asian	6 (50.0%)
Experience and expertise	
Country of practice	
The United Kingdom	8 (66.6%)
The Netherlands	4 (33.3%)
Years working with IBD patients, mean (SD)	11.0 (9.3)
Profession	
Nurse	4 (33.3%)
Gastroenterologist	8 (66.6%)
Sees IBD patients exclusively	5 (41.6%)
Sees IBS patients	6 (50.0%)

### Theme I: focus on disease activity, not pain and associated symptoms

This theme discusses the practice of assessing and managing pain (and associated symptoms) during remission and is broken down into two subthemes: (1) *Diagnostics: assurance by objective measures* and (2) *Treatment: treating disease, not pain and symptoms*.

#### Diagnostics: assurance by objective measures

HCPs mentioned that they concentrate on parameters of active disease in the first instance – they consider these to be most concerning – and that they have insufficient time and resources to attend to symptoms that are of limited consequence to the disease.

− *‘I have 15 minutes […] I usually start with the SCCAI or the Harvey Bradshaw, I want to know weight, I discuss lab results, including the calprotectin test, possible other investigations. I look up when they are due another surveillance. Then I go through the medication and possible side effects. [15 minutes are] filled soon enough […] and then there is a little bit of room for […] social talk left’. (Gastroenterologist, P07)*.

They generally adhere to diagnostic criteria, which do not necessarily include pain.

− *‘We adhere to the guidelines as much as we can […] we have a set of questions we ask […] that does not even include abdominal pain as symptom […] because abdominal isn’t specific enough to establish elevated disease activity [in colitis]. It’s different for Crohn’s, the Harvey Bradshaw Index […] does address abdominal pain’. (Gastroenterologist, P07)*

HCPs confessed that they prefer to focus on active disease and objective biomedical tests. They feel less at ease saying that symptoms are functional.

− *‘I think as I’ve become more and more experienced, I think I’m less likely to [say that symptoms are functional…] my knowledge has increased and also, I’ve been lucky enough to be at centres where I can do some of these specialist tests […] I’m less likely to be just as dismissive’. (Gastroenterologist, P08)*

An aspect that might be relevant to this sense of unease is that pain and symptoms that cannot be explained by disease are less controllable and predictable.

− ‘*Pain that isn’t caused by inflammation is […] quite abstract and quite difficult to pin down […] I mean we acknowledge that pain is what the patient says it is, but there is not that much out there on pain […] it is really hard and I don’t have an answer’. (IBD-nurse, P02)*

#### Treatment: treating disease, not pain and symptoms

When HCPs found that test results pointed to active disease, they would start treatment to reduce inflammation and aid mucosal healing. Symptoms, such as pain, were presumed to subside in parallel with inflammation and generally not specifically targeted.

− *‘We are not always treating pain as an independent thing; we are treating pain as part of the whole inflammatory bowel disease process’. (IBD-nurse, P06)*

Treating symptoms in the absence of inflammation was perceived to be more challenging because of limited management options. One gastroenterologist compared treating pain in remission to *shooting an arrow in the dark* and said that *even the consultants with much longer experience* struggle with it *(Gastroenterologist, P09)*. He observed that he did not always find it easy to confront the topic of pain during consultations.

− *‘I tend to avoid the topic if the patient does not bring it up. […] Unless they tell me they have pain, I will ask it as a do you have any abdominal pain? And if no, I will leave it at that’. (Gastroenterologist, P09)*

The limited management options could lead to feelings of inadequacy.

− ‘*I still need to do something about it and I can’t just discharge them […] I couldn’t do that with my own conscience to just discharge them […] I’d feel bad not to be able to try and offer something’. (Gastroenterologist, P08)*

Some HCPs reported benefits of referring patients to specialist psychologists, dietitians, or psychiatrists, indicating that perhaps the management of chronic pain was seen as out of scope for gastroenterologists and IBD-nurses.

− ‘*If someone has got pain then the only thing you can really do is refer to a psychologist or a dietician […] after that you are out of options really. I think gastroenterologists need better ways of dealing with it really’. (Gastroenterologist, P11)*

Still, most HCPs thought that giving explanations for ongoing pain (and potential other symptoms) was important and offered them. Unfortunately, with little provisions available, this appeared to be courtesy and not standard practice, the impact of which will be described in further detail below.

### Theme II: idiosyncratic and uncertain explanations for pain during remission

Limited resources and time meant that addressing and explaining pain during remission relied on the personal values, experience, and expertise of HCPs. We found variations in the degree to which HCPs valued a personal connection with patients, offered pastoral care, or felt the need to offer relief or reassurance. One HCP reflected on how the care she provided had been influenced by her personal needs over the course of her practice.

− *‘But we did investigate the life out of it, and I sat her down and I said look, I cannot find anything, we have done everything, what I can tell you is there is definitely no cancer in there, there is definitely no active Crohn’s disease in there, I said, but I have not got an answer for it, and I am going to stop looking for it now. And she said fine,* that is *all I needed to know. Changed the way I practice because I felt I had to give her an answer’. (IBD-nurse, P06)*

One of the nurses struggled to address ongoing pain as she did not see her need to offer relief and education reflected in the wider IBD team.

− *‘Your consultant would not look after that pain […] it is not part of standard operation, we know about it, but we do not really do anything different. So mentally I panic because I think I do not know what I am doing. […] feel so bad because even my colleagues, who have got 10 more years of experience on me, I feel like their response is not what I expect it to be’. (IBD-nurse, P03)*

These quotes illustrate the self-perceived, nuanced role that experience, expertise, and profession (nurse versus gastroenterologist) play in pain/symptom management. A lack of clear guidelines in the area seemed to have fostered a high variety in, and uncertainty of, explanations. We generated two subthemes addressing this: (1) *IBS in quiescent IBD, in-between diagnosis and explanation* and (2) *Uncertainty and variability of explanations*.

#### IBS in quiescent IBD, in-between diagnosis and explanation

While some HCPs reported adhering to the Rome IV criteria, others reported discussing secondary IBS with patients even when they did not strictly meet the criteria for an IBS diagnosis; they found it a useful explanatory model because of the familiarity of patients with it.

−*‘I think that everyone pretty much knows what IBS is. So yeah, I do think it is helpful to call it that, even though not everyone will meet the criteria for it’. (Gastroenterologist, P05)*

IBS seems to have acquired the ambiguous status of something between a diagnosis and an explanation. This was exemplified by the fact that HCPs inconsistently reported secondary IBS in patient notes.

− *‘I might suggest to someone that their symptoms are caused by this brain-gut axis dysfunction, or whatever, but I still think calling it IBS is still another extra step […] a proper diagnosis that stays on the records forever’. (Gastroenterologist, P11)*

Moreover, different labels were used to indicate IBS. While HCPs explained that they are careful with labels as these might be perceived as dismissive by patients, they did not necessarily agree on which labels were preferable. One gastroenterologist consistently spoke of functional symptoms, which others found dismissive.

− *‘I don’t like using the word functional pain or a functional problem because I think it sounds quite dismissive’. (IBD-nurse, P02)*

Finally, one HCP mentioned that IBS in IBD might be a separate entity and explanations needed to be adapted accordingly.

− *‘… patients who are in remission still can have abdominal pain in a chronic form which can be seen as irritable bowel syndrome. But of course, in IBD the question is whether* that is *a different entity based on long standing inflammatory processes that have been there previously and are now then leading to visceral hypersensitivity’. (Gastroenterologist, P16)*

#### Uncertainty and variability of explanations

We found a high variety and uncertainty in explanations for ongoing pain. Table 3 gives an overview of the different explanations (categorised to illuminate their variety) and highlights expressions of uncertainty (underlined). One IBD-nurse reported that she struggled to explain pain during remission and expressed a need for more guidance; she had little confidence in the explanations she was providing and was unsure where to draw a line or how to adapt her explanation to a patient.

− *‘… we might talk about the anatomy and* that is *all I talk about*. *It is a bit made up to be honest’*. *(IBD-nurse, P03)*

**Table 3.** Healthcare professionals’ explanations of ongoing symptoms and pain during remission.

Normalisation	
1.	*‘That is IBS. Those are functional symptoms. I would then say that [the intestines] are a bit upset and that that is very common, also in people who do not have Crohn’s disease or ulcerative colitis and that it, because it is so common, sometimes also presents with Crohn’s disease or ulcerative colitis’. (Gastroenterologist, P07)*
Irritation of anatomical structures	
2.	*‘I do not know if it is the right thing, but I try and explain it to them it is really inflamed, if you imagine like if you scraped your leg then it is very ulcerated and swollen and irritated and then essentially if poo is passing past that then of course it is going to exacerbate it. I think that is it on a very basic level, but I think some of them do not get that that is why it happens’. (IBD-nurse, P02)*
Inflammation and sensitivity	
3.	*‘There are studies [showing] that patient with an irritated bowel, if a balloon [is introduced and] is inflated, that will literally be felt sooner by people with an irritated bowel than by people without an irritated bowel’. (Gastroenterologist, P05)*
4.	*‘The hypersensitivity word always comes up a lot when you talk about this sort of thing, if your bowel is hypersensitive then we often can’t find the cause on a scan because it’s at a microscopic level but lots of people have hypersensitive guts and so yours is just more sensitive [than] other people’s and so you will react differently to certain things that other people won’t react to and that’s why you are getting pain or diarrhoea and things’. (Gastroenterologist, P11)*
5.	*‘I think there is some* […] *scientific evidence, although it is not hard *[*evidence*]*, that an intestine that has been inflamed has more pain receptors. So, that is something people like to hear, that it is not between their ears’. *(*Gastroenterologist, P05*)
Remodulation enteric nervous system	
6.	*‘Uh, uh, perhaps I sometimes mention that perhaps your bowel is a little bit more primed to feeling things because you have had inflammation in the past. I do not know how scientifically that is, but* […] *it certainly sounds plausible’*. (*Gastroenterologist, P08*)
7.	*‘Then you start setting expectations that, look, your gut may never be normal *[…]* it is going to be hypersensitised, *[…]* the wiring in the nervous system of the gut is different, it changes, it is remodulated. As a result, your gut behaves differently’. *(*Gastroenterologist, P12*)
Gut emotions	
8.	*‘I always say that there are more nerves in the gut than everywhere else other than the brain *[…]* the brain feels things like anxiety, depression, and stress *[…]* the gut is not conscious it does not have those symptoms *[…]* it does have *[…]* nausea, pain, diarrhoea, that sort of thing. So, when the gut feels stressed or anxious *[…]* the symptoms you get are manifested as diarrhoea and pain and things’. *(*Gastroenterologist, P11*)
Microbiome	
9.	*‘Or we talk about microbiome, and I say look I think your bacteria could have been altered and [your] microbiome can be changed over the years with all the inflammation’. (Gastroenterologist, P12)*
Brain-gut axis	
10.	*‘So, I had a diagram with me, and I said OK this is the bit of the bowel that is inflamed, now in the abdomen it is not like your finger where you cut your finger and your brain says this is where the pain has come from because you have got an injury here. In your abdomen the nerves are spread all around and the traffic system is not that good, so the road map is not that good. So often your brain cannot quite localise where that pain is so that is why your pain is not necessarily where your inflammation is’. (Gastroenterologist, P09)*

Explanations were given in the context of IBS, or in isolation, and could refer to basic statistics (*explanation 1*), describe how sensitivity manifests (*explanation 3*), or delineate mechanisms (*explanation 7*). Some HCPs would adapt explanations to specific symptom presentations or the person in front of them.

HCPs relied on their own common-sense *(explanation 6)*, reading *(explanation 5)*, and chance conversations to shape their explanations which likely has contributed to the uncertainty and wide variation in explanations.

− *‘I’d recently seen a talk, or spoken to some people about pain and the psychology of abdominal pain and that sort of thing, so I though let me try this out on her, and she wasn’t the right one to try it out on […] she wasn’t having it at all’ (Gastroenterologist, P11)*

Different clinical experience also seemed to add to the variation in explanations.

− *‘I think [palliating/understanding pain during remission] is a little bit more difficult for them [nurses] and often the consultant at the end of the day, because they might be using a more biomedical model of care rather than a biopsychosocial model […]. Their viewpoint might be slightly different to mine because I come at it from a slightly different angle because I run the IBS clinic as well’. (Gastroenterologist, P10)*

Most HCPs presented an integrated view of pain, which was particularly clear in those who included the role of physiological pathways and the brain in their explanations (see example 10, Brain-Gut Axis, in table 3).

### Theme III: shared decision-making versus shared sensemaking

That HCPs endeavoured to provide explanations while sometimes feeling ill-equipped to do so, may be a consequence of the benefits that explanations offer. Firstly, explanations have the potential to reassure people and help them cope with ongoing pain (and related symptoms).

− *‘About 50% of the patients finds [pain education] enough, so, do not want or need any treatment for their pain afterwards, they just have some worries […] and if you can explain to them that the inflammation is not present […] and that pain is due to previous inflammation, or whatever, that for many patients is already enough and that they can deal or cope with the pain themselves’. (Gastroenterologist, P16)*

Secondly, explanations may form the basis for shared decision-making which is defined as ‘an approach where clinicians and patients share the best available evidence when faced with the task of decisions, and where patients are supported to consider options, to achieve informed preferences’.^21,22^

− *‘The other group still says OK […] I can rearrange my thoughts around that, but still, I have pain so I can’t go to work, or I have pain and I can’t go to this party […] studies are lacking on what’s the best treatment […] at the end, the best treatment for those patients is the treatment that they find most suitable for themselves’. (Gastroenterologist, P16)*

#### Shared decision-making

The extent to which HCPs adopted shared decision-making and helped patients to consider options differed. Some HCPs reported to have some kind of *‘spiel’* explaining ongoing pain *(Gastroenterologist, P11)* or used objective measures as a means to both reassure and convince people.

− *‘That we have gone in [endoscopically] and have seen that it is normal also reassures them […] and you tend to find that they buy in to the whole concept that this pain is probably more functional in its cause’. (Gastroenterologist P09)*.

Additionally, when HCPs found it hard to establish common ground, they might fall back on second opinions and additional tests to re-establish trust.

− *‘If you, as doctor, are only deflecting and reassuring the patient, you will at some point lose their trust, and to be able to ask for a second opinion […] is very welcome then. Just as a verification that you are right…’ (Gastroenterologist, P07)*

#### Shared sensemaking

HCPs reported more collaborative efforts to make sense of symptoms as well. For instance, some HCPs helped people by guiding them through the diagnostic process or teaching them how to appraise their pain.

− ‘*I predominantly see myself as an advisor, a sort of coach, so I very much try to brainstorm with people [and ask:] What type of pain? When does it occur? Does it hinder your daily life at work with sports? Intimacy? […] I am no psychologist […], but with some patients I might seek that boundary’. (Gastroenterologist, P05)*

Additionally, while most HCPs infer secondary IBS/functionality after excluding organic causes for pain, two neurogastroenterologists reported making positive IBS diagnoses ahead of investigations.

− *‘So often people are told all your tests are normal, it must be IBS, and* that is *a very negative way of doing it […] you can positively diagnose it based on […] pain related to defecation, abdominal distension, or bloating [… which] would allow me to say up front to the patient I think you have IBS, however I would like to rule out active inflammation […] my assessment is guiding me as to which of the three directions. One is more neuropathic, one is more IBS treatment, and the other one is Crohn’s or colitis treatment. So, if I just relied on the tests, it would only give me one answer, is there active Crohn’s or not, is there active colitis or not. But it will not help me treat the pain which is a symptom’. (Gastroenterologist, P10)*

One of them additionally reported that it is important to adapt explanations to the concerns of the individual patient.

− *‘… some patients explain […] that some foods trigger their symptoms, some explain more about their stress […] anxiety or depression or previous trauma come up […] I explain about the gut brain interaction and tailor that explanation on the symptoms that they have just told me […] I can explain them the whole pathophysiology of irritable bowel syndrome […and] IBD, but if they do not find themselves in that story, then it is just the doctor talking too much’. (Gastroenterologist, P16)*

In summary, while shared sensemaking requires an initial time investment, this was generally perceived to pay dividends later; HCPs felt that patients felt less worried and were more receptive to a secondary IBS diagnosis.

## Discussion

This study explored how HCPs manage patient reports of abdominal pain and IBS-like symptoms during quiescent IBD. Reflective thematic analysis revealed that HCPs sometimes struggle to address pain and symptoms when there is no clear underlying pathology, and prefer to concentrate on objectifiable causes for symptoms and treating disease activity *(theme I)*. We found that explanations of pain during remission are not standard *(theme II)* and described how HCPs incorporate shared decision-making and shared sensemaking in their daily practice *(theme III)*. In this discussion, we will provide a context for these findings and make some recommendations.

Results showed that HCPs do not specifically target symptoms and prefer to focus on reducing inflammation as symptoms (including pain) generally diminish in parallel with the inflammation. Similarly, HCPs reported predominantly relying on objective biomedical tests to explain symptoms. Active disease obviously commands urgent action, there are long term benefits to tight control, and a focus on biomarkers aligns with recent therapeutic goals shifting from symptom control to mucosal healing.^23,24^ However, disease management is often insufficient to treat pain and other symptoms^2,11,25,26^ and evidence shows that people’s quality of life is impacted when pain is insufficiently addressed.^27–29^ A prospective survey amongst 197 HCPs providing IBD care reported that approximately 21% of them feel uncomfortable addressing pain.^30^ Our findings indicate that this might due to subjectivity of pain, the limited tools to deal with it, and the fact that symptom management may not be considered central to the role of disease experts (potentially resulting in an overuse of second opinions and additional tests). Overreliance on biomedical tests may be problematic as multiple testing and consultations can heighten distress and have iatrogenic consequences.^12^

Good information and education may help patients face the medical, emotional, and social challenges posed by chronic diseases,^31^ increase patients’ potential to adjust to their IBD,^32^ and increase health literacy thereby advancing people’s ability ‘to gain access to, understand and use information in ways which promote and maintain good health’.^33^ Explanations in our study were highly variable and not always tailored to individuals: they might be complex and address cognitive reassurance (the brain-gut axis), but could also be simple and focussed on affective reassurance (IBS is common in people with IBD).^34^ Explanations focussed on former type of reassurance are reported to be more effective in reducing distress and healthcare utilisation,^34,35^ Moreover, flexible explanations – adapted to both illness and patient – foster self-determination and promote that both lived and clinical experience inform decisions (which both are critical to shared decision-making,^21^ a key priority of the National Health Service; NHS).^36^ Unfortunately, in line with previous findings,^30,37^ our study suggests that explanations were used to reassure/convince patients rather than to move towards informed decision making.^22^

Most HCPs inferred secondary IBS/functional symptoms after they excluded organic causes for pain and relayed this to patients. However, diagnostic criteria for IBS were applied loosely – potentially leading to inflation of the term – and the label might be perceived as dismissive. While it has been proposed that clinical encounters should focus more on explanations than diagnoses,^38^ a proportion of people with ongoing symptoms has been reported to benefit from a secondary IBS diagnosis.^3^ It is worth considering whether more patients would respond favourably to the suggestion of IBS if it was framed differently. A recent study found that different diagnostic labels for low back pain influence both patients’ perceptions of threat and treatment intentions, thereby showing how important it is to check the way patients understand labels and judge implications.^39^

Interestingly, HCPs who made positive IBS diagnoses early in the diagnostic trajectory, reported high acceptance of IBS explanations. Earlier research has suggested that patients with chronic illnesses better understand their condition when HCPs incorporate explanations in their history-taking process.^40^ Early explanations – that is, shared sensemaking – not only aids understanding, they also allow HCPs to teach by example and help patients assess whether pain and other symptoms are indicative of active disease or not. As such it has the potential to enhance health literacy,^41^ improve outcomes, and reduce health care costs^42^; it may require an initial time investment but has the potential to repay this later.

### Strengths and limitations

This study had several limitations. Firstly, interviews were conducted with Dutch and British HCPs and findings may be restricted to healthcare settings in respective countries. However, healthcare in the United Kingdom and the Netherlands is organised and funded in different ways – covered by an insurance versus state funded – and we found no obvious differences in how HCPs managed and explained pain and symptoms during remission. Secondly, pressures on the healthcare system due to COVID-19 meant that we were unable to recruit a larger more diverse population. Still, interviews lasted sufficiently long to go into depth and participants varied in experience level, interest in IBS, occupation, gender, and ethnicity. Furthermore, we achieved variety and density when these were appropriate and we feel we approached thematic saturation.^16,17^ Thirdly, due to the nature of our research, we could not verify what happens in the consultation room when HCPs offer explanations. This meant that we could not verify the variation in explanations, either with-in or between HCPs. We could neither assess how effective different strategies and explanations were, nor why they were effective or ineffective. Fourthly, one of our participants reported that HCPs differed in how they established and evaluated remission. Such differences presumably affect how explanations are construed and – more importantly – how they are received. This would be of interest to explore, but our research design was not set up for this. Finally, we acknowledge that different HCPs – such as general practitioners, psychologists, pain specialists, dieticians, and physiotherapists – have different approaches to managing and communicating pain. It would be beneficial to investigate how these parties view IBD pain management and communication to further explore and streamline explanations.

### Implications for future research and clinical practice

Based on our findings, we suggest that an expert panel, including a multidisciplinary team of HCPs who provide care to people with IBD (e.g. gastroenterologists, nurses, psychologists, and dieticians) and patients with IBD, should be brought together to develop an initial agreed working definition of (a) how to define pain and symptoms during remission, (b) when to diagnose IBS in the context of IBD, and (c) how to explain both of these to patients. Ensuing recommendations should lead to the development of evidence-based explanations that help guide discussions with patients and introduce shared sensemaking and shared decision making into practice. These explanations should be easily adaptable to specific symptom presentations and different health literacy levels, and may potentially be supported by pamphlets, online resources,^43^ and multidisciplinary care teams.^44^

Explanations may be informed by what we already know from pain education. Pain education has been shown to improve important clinical variables, including pain and disability, by reconceptualising pain as an alarm signalling the need to protect body tissues from (further) damage.^45^ Explanations most valued by chronic pain patients integrate messages that (a) pain does not necessarily mean underlying tissue damage, (b) thoughts, emotions, and experiences affect pain, and (c) that pain can become overprotective (raising the alarm when there no longer is a need), but that doesn’t mean it is not modifiable.^46^ Explanations and education in the context of quiescent IBD may include the brain-gut axis, central processing of pain, and/or hypersensitivity,^3^ all of which can be delivered within the context of secondary IBS, or in isolation (people who do not strictly fulfil the criteria for IBS may benefit from similar explanations^47^). Additionally, as already alluded to by some of the HCPs, it will be important to validate patients’ experiences^48^ and acknowledge both that pain is real and may induce uncertainty. ^3^ It will furthermore be critical to consider what patients understand to constitute a flare or remission.^14^ Any communication should consider the patient’s illness history and level of health literacy.^3^

Mentioning IBS as a potential diagnosis early on and adapting explanations to patients’ own understanding of their specific complaints,^49^ might be strategies that are more acceptable, improve understanding, and promote positive outcomes.^32^ However, all explanations would need to be accompanied with clear treatment and management guidance. At a more macro level, ongoing issues around service constraints with regards to psychosocial support and behavioural treatment for managing symptoms should be addressed.

### Conclusions

We showed that IBD care relies largely on objective biomedical tests to explain symptoms, primarily focuses on treating disease activity, and that explanations of pain and other symptoms during remission are not standard. Future work should focus on the formulation of standardised explanations for ongoing pain and symptoms to aid HCPs adopt shared sensemaking and shared decision making in their practice. Explanations should be easy to adapt to different health literacy levels and may be supported by online resources as many patients consult these.
